# Lifeomics leads the age of grand discoveries

**DOI:** 10.1007/s11427-013-4464-6

**Published:** 2013-03-23

**Authors:** FuChu He

**Affiliations:** State Key Laboratory of Proteomics, Beijing Proteome Research Center, National Center for Protein Science (Beijing), Beijing, 100850 China

**Keywords:** age of grand discoveries, lifeomics, life sciences

## Abstract

When our knowledge of a field accumulates to a certain level, we are bound to see the rise of one or more great scientists. They will make a series of grand discoveries/breakthroughs and push the discipline into an ‘age of grand discoveries’. Mathematics, geography, physics and chemistry have all experienced their ages of grand discoveries; and in life sciences, the age of grand discoveries has appeared countless times since the 16th century. Thanks to the ever-changing development of molecular biology over the past 50 years, contemporary life science is once again approaching its breaking point and the trigger for this is most likely to be ‘lifeomics’. At the end of the 20th century, genomics wrote out the ‘script of life’; proteomics decoded the script; and RNAomics, glycomics and metabolomics came into bloom. These ‘omics’, with their unique epistemology and methodology, quickly became the thrust of life sciences, pushing the discipline to new high. Lifeomics, which encompasses all omics, has taken shape and is now signalling the dawn of a new era, the age of grand discoveries.

Natural science is the “art” of discovery, and discovery is the cornerstone of natural science. Discovery, which explores the known and the unknown, is a mastery that requires accumulated knowledge and original innovation. A detailed look at the history of science reveals numerous examples of ‘spurring with long accumulation’: when our understanding of a field accumulates to a certain level, we are bound to see the rise of one or more groundbreaking scientists. These great scientists will trigger a flood of discoveries, break the shackles of time and advance our understanding to new high. The result is an explosive growth in one or more related disciplines, bringing new ideas and fundamental changes and this is when a new era begins, i.e., the age of grand discoveries. Thanks to the rapid development of molecular biology over the past 50 years, especially in the area of omics over the past 20 years, contemporary life science is once again approaching its breaking point and the trigger for this is most likely to be ‘lifeomics’ (i.e., genomics, RNAomics, proteomics, etc.).

## 1 The age of grand discoveries in science

Our understanding and learning of natural sciences begins with mathematics. Modern science is precisely the pursuit of mathematical laws governing nature. Indeed, many scientific breakthroughs were made through the development of mathematics. The Pythagoreans of ancient Greece made remarkable contributions that triggered the first age of grand discoveries in mathematics. They were the earliest people to demonstrate the Pythagorean Theorem and propose the concepts of odd, even, prime, amicable and whole numbers. The Pythagoreans considered arithmetic as an ‘absolute and discrete quantity’, music as a ‘relative and discrete quantity’, geometry as a ‘static and continuous quantity’, and astronomy as a ‘dynamic and continuous quantity’. They established the doctrine that ‘mathematics is the basis of everything’. Because of this doctrine, the Greek philosopher Plato wrote above his academy entrance, “let no one ignorant of geometry enter here.” He viewed mathematics as an indispensable tool for understanding the structure of the universe.

Thereon, the development of mathematics became the cornerstone of all disciplines that followed.

The age of grand discoveries in geography lasted a mere 40 years, but its impact on the world was felt for hundreds of years. At the turn of the 16th century, the geocentric theory and the European compass took us to places and routes unknown to civilization. In 1485, Christopher Columbus discovered North America; in 1497, Vasco da Gama discovered the Indian Ocean and India; in 1498, Christopher Columbus discovered South America; in 1519, Ferdinand Magellan sailed to the southern tip of South America and discovered a strait that joins the Atlantic and Pacific Oceans; and in 1521, Magellan crossed the strait and discovered the Pacific Ocean, marking a major turning point in modern Western civilization [1]. These geographical explorations brought forth a spread of revolutionary ideas and commercial activities, greatly expanding the universe as conceived by Aristotle and Ptolemy. The change prompted many intellectuals in Europe to question the authority of the Church, challenging their age-old beliefs and views about the world. The resulting awakening opened the door to critical thinking and promoted innovation in the revolution of science.

Chemistry experienced immense growth during its founding period thanks to the discovery of gases and elements in the 18th century. In 1756, Joseph Black discovered carbon dioxide; in 1760, Daniel Rutherford discovered nitrogen; in 1766, Henry Cavendish discovered hydrogen; and in 1774, Joseph Priestley discovered oxygen [1]. As a result of these discoveries, in 1789, Antoine-Laurent Lavoisier provided a more scientific explanation for combustion—in terms of oxidation—to challenge the then prevailing phlogiston theory. After extensive research, Lavoisier wrote the book *Traité Élémentaire de Chimie* (*Elementary Treatise on Chemistry*) and presented his definition of an element. Because of his work, more than half of all elements that exist in our universe for billions of years were found in the decades that followed (Table 1). It was nothing more than a glory for human beings. In 1869, Dmitri Mendeleev organized the then known 63 elements into groups with similar properties. He recognized that there was a pattern in the way elements behave and created the first periodic table. The examples above illustrate how some of the greatest discoveries in chemistry were gestated through accumulation or evolution of knowledge and finally born from their revolution.

So far, we have only mentioned disciplines whose ages of grand discoveries took place at the early stage of development. People might ask, "can a mature discipline enter the age of grand discoveries once again?" The answer is yes, and astronomy and physics are two great examples.

Astronomy and mathematics are the oldest of the natural sciences. These disciplines spearheaded the development of science during the Age of Enlightenment. The emergence of heliocentric theory and telescope technologies at the turn of the 17th century triggered the first age of grand discoveries in astronomy. In 1572, Tycho Brahe detected a stellar explosion in the constellation Cassiopeia. He made detailed observations and records for 16 months before reporting the discovery of a supernova. In 1577, Tycho studied a bright comet for 74 days and, based on his observations, deduced that our planet was orbiting the Sun and that comets were not atmospheric phenomena [1]. In 1576, Tycho received the financial support from Frederick II, the then King of Denmark, to build an observatory on the island of Hven. Since then, Tycho had been studying the movement of stars, planets, the Sun and the Moon, day after day, year after year, for 21 years. He collected a huge amount of observational data before publishing his first comprehensive star catalogue, which detailed the position of more than 1000 stars. Known as the 'king among astronomers', Tycho pushed observational astronomy to new high using naked eyes only. His celestial positions were amazingly accurate, superseding those of any predecessor. In 1600, Tycho offered the talented Johannes Kepler unrestricted access to his wealth of observational data. Based on Tycho's precise data, Kepler worked out the orbits of the planets and developed his three laws of planetary motion. He showed that all planets move in ellipses with the Sun at one focus, proving that the Copernicus' heliocentric model was indeed correct. Because the planets of our solar system move through space according to Kepler's laws, many people called Kepler the 'legislator of the heavens'. Even Kepler's tomb carries an epitaph he wrote himself, "I measured the skies, now the shadows I measure. Skybound was the mind, earthbound the body rests". Some say it was only because of Tycho's precise observations that Kepler worked out the laws of planetary motion. Therefore, for a mature discipline such as astronomy to enter the age of grand discoveries again, it must first accept a revolutionary hypothesis. Subsequently, discoveries made in the new age can be used to support the hypothesis.

The early 17th century saw the birth of another physicist whose achievements equalled those of Kepler—Galileo Galilei. He is considered by many to be the 'father of science', as well as the creator of modern experimental science. In 1604, Galileo derived the first (uniform velocity) and second laws of motion (uniform acceleration); in 1609, he discovered uneven terrains on the moon and created a topological map of the lunar surface using a telescope he made for observing celestial bodies [1]; Galileo discovered Jupiter's four satellites, providing supportive evidence for Copernicus' theory; two years later, Galileo discovered sunspots, solar rotation and the planetary phases of Venus and Mercury. The chain of discoveries initiated a new age of grand discovery for astronomy. Galileo established a research approach that integrates experimental measurements with the- oretical analyses through observations. His famous quote, "measure what can be measured, and make measura-

**Table 1** Chemical elements that were discovered in the 19th century (52 different elements)

| Z | Year | Chemical element | Country | Discovered by |
|---|---|---|---|---|
| 1 | 1868 | He (Helium) | France | Pierre Janssen |
| | | | UK | Named by Lockyer S. N. |
| 2 | 1817 | Li (Lithium) | Sweden | Arfwedson J. A. |
| 3 | 1808 | B (Boron) | UK | Davy H. |
| 4 | 1898 | Ne (Neon) | Germany | Koch Robert |
| 5 | 1807 | Na (Sodium) | Germany | Davy H. |
| 6 | 1808 | Mg (Magnesium) | France | Davy H. |
| 7 | 1825 | Al (Aluminium) | Denmarl | Oeisted H. C. |
| 8 | 1823 | Si (Silicon) | Sweden | Bertholius J. J. |
| 9 | 1810 | Cl (Chlorine) | Sweden | Scheele C. W. |
| | | | UK | Named by Davy H. |
| 10 | 1894 | Ar (Argon) | UK | Rayleigh J. W. & Ramsay W. |
| 11 | 1807 | K (Potassium) | UK | Davy H. |
| 12 | 1808 | Ca (Calcium) | UK | Davy H. |
| 13 | 1879 | Sc (Scandium) | Sweden | Nilson L. F. |
| 14 | 1831 | V (Vanadium) | Sweden | Sefstrom N. G. |
| 15 | 1875 | Ga (Gallium) | France | De Boisbaudran L. |
| 16 | 1885 | Ge (Germanium) | Germany | Winkler C. A. |
| 17 | 1817 | Se (Selenium) | Sweden | Berzelius J. J. |
| 18 | 1824 | Br (Bromine) | France | Balard A. J. |
| 19 | 1898 | Kr (Krypton) | UK | Ramsay W. |
| | | | UK | Named by Travers M. W. |
| 20 | 1860 | Rb (Rubidium) | France | by Bunsen R. |
| | | | | Named by Kirchhoff G. R. |
| 21 | 1808 | Sr (Strontium) | UK | Davy H. |
| 22 | 1801 | Nb (Niobium) | UK | Hatchett C. |
| 23 | 1827 | Ru (Ruthenium) | Russia | Klaus K. K. |
| 24 | 1803 | Rh (Rhodium) | UK | Wollaston W. H. |
| 25 | 1803 | Pd (Palladium) | UK | Wollaston W. H. |
| 26 | 1817 | Cd (Cadmium) | Germany | Stromeyer F. |
| 27 | 1863 | In (Indium) | Germany | Richter H. T. & Reich F. |
| 28 | 1814 | I (Iodine) | France | Courtois B. |
| 29 | 1898 | Xe (Xenon) | UK | Ramsay W. |
| | | | | Named by Travers M. W. |
| 30 | 1860 | Cs (Caesium) | Germany | Bunsen R. & Kirchhoff G. R. |
| 31 | 1808 | Ba (Barium) | UK | Davy H. |
| 32 | 1839 | La (Lanthanum) | Sweden | Mosander C. G. |
| 33 | 1803 | Ce (Cerium) | Sweden | Berzelius J. J. |
| | | | Germany | Klaproth M. H. |
| | | | Sweden | Hisinger W. |
| 34 | 1885 | Pr (Praseodymium) | Austria | Auer von Welsbach C. |
| 35 | 1885 | Nd (Neodymium) | Austria | Auer von Welsbach C. |
| 36 | 1879 | Sm (Samarium) | France | De Boisbaudran H. L. |
| 37 | 1896 | Eu (Europium) | France | Demarcay E. |
| 38 | 1880 | Gd (Gadolinium) | Sweden | De Marignac J. G. |
| | | | France | First isolated by de Boisbaudran H. L. |
| 39 | 1843 | Tb (Terbium) | Sweden | Mosander C. G. |
| 40 | 1886 | Dy (Dysprosium) | France | De Boisbaudran H. L. |
| 41 | 1879 | Ho (Holmium) | Sweden | Cleve P. T. |
| 42 | 1843 | Er (Erbium) | Sweden | Mosander C. G. |
| 43 | 1879 | Tm (Thulium) | Sweden | Cleve P. T. |
| 44 | 1878 | Yb (Ytterbium) | Switzerland | Marignac J. G. |
| 45 | 1802 | Ta (Tantalum) | Sweden | Ekaberg A. G. |
| 46 | 1803 | Os (Osmium) | UK | Tennant S. et al. |
| 47 | 1803 | Ir (Iridium) | UK | Tennant S. et al. |
| 48 | 1861 | Tl (Thallium) | UK | Crookes W. |
| 49 | 1898 | Po (Polonium) | France | Curie P. & Curie M. S. |
| 50 | 1898 | Ra (Radium) | France | Curie P. & Curie M. S. |
| 51 | 1899 | Ac (Actinium) | France | Debierne A. L. |
| 52 | 1828 | Th (Thorium) | Sweden | Berzelius J. J. |

ble what cannot be measured", has inspired many experimentalists for dozens of generations. Galileo established a new direction for theoretical physics on the basis of experimental science. Because of him, we have boundless freedom to explore the physical world.

Discoveries made by Kepler and Galileo ushered in a new era—the Newtonian era. Issac Newton invented the binomial theorem and differential calculus in mathematics, established the law of universal gravitation in astronomy, laid down the three laws of motion in physics, and discovered the visible spectrum and reflecting telescope in optics. Having made these great achievements, Newton in 1686 published *Philosophiae Naturalis Principia Mathematica* (*Mathematical Principles of Natural Philosophy*) [1], a masterpiece that epitomizes his work on mechanics and gravitation. Lord Newton once said, "if I have seen further it is by standing on the shoulders of giants". Many people take these as Newton's humble words, but if we review them carefully, we realise the true meaning of his words. It was through Tycho's precise data that Kepler derived the laws of planetary motion; it was through large amounts of experimental work that Galileo derived the first and second laws of motion; and it was through the integration of related laws in physics and astronomy that we arrived at the epitome of Newtonian mechanics. In other words, Newton understood that it was only through standing on the shoulders of giants like Tycho, Kepler and Galileo that he achieved so much in his lifetime. In the same sense, it was through large amounts of observation, accumulation of experimental data, and grand discoveries of laws that we produced these giants.

In the late 19th century, the theoretic building of Newton epitomized the studies of acoustics, optics, electromagnetism and thermodynamics. His 'universal' laws worked on all matter, from the smallest of microscopic particles to the largest of heavenly bodies. People felt there were little room for expansion in the development of science. However, there were two 'dark clouds' hanging over classical physics: the Michelson-Morley experiment and blackbody radiation. Thanks to these two phenomena, relativity and quantum theory were developed to replace Newtonian mechanics as the more generalized theories of the physical world. In addition, a new 'hypothesis-driven' research approach, as opposed to the conventional "discovery-driven" research approach, emerged. Therefore, can we derive all theories from hypotheses? The answer is not always, even for highly theoretical disciplines like astronomy and physics where theories are typically derived from observations. Some of these observations rely on hypotheses, while others have completely no theoretical bases. For example, discoveries derived from aforementioned geocentric and heliocentric theories were driven by and proven from hypotheses. However, the discovery of Kepler's three laws and Galileo's two laws did not rely on any hypothesis. They were purely based on scientific observations and vast amounts of scientific data. Even in the case of relativity, which involves a lot of thought experiments, the main assumptions were based on results from the Michelson-Morley experiment. In reality, the hypothesis-driven approach is just as important as the discovery-driven approach when it comes to developing science and technology. Like what the pioneer Galileo once said, to "measure what can be measured, and make measurable what cannot be measured." This should be the basic principle of modern science, especially experimental science.

In light of these examples, we see that only through accumulation of knowledge can we get results. Discovery is a process led by theory and driven by technology. Without discovery, there will be no scientific revolution.

## 2 The age of grand discoveries in life sciences

Life science is an ancient but exciting discipline where new discoveries are being made everyday. Its golden era, as a whole, spans from 500 years ago to the present.

Modern life science was triggered by great discoveries in biology during the 16th and 17th centuries. In 1543, the Flemish anatomist Andreas Vesalius gained a thorough understanding of the human anatomy through dissecting animals and human cadavers. His book *De Humani Corporis Fabrica* (*On the Human Body Structure*) gave detailed descriptions of the human body, including its skeletal system, muscular system, vascular system, nervous system, digestive organs, internal organs and sensory organs. The discovery was a major triumph over ignorance and Christian supremacy. The book *De Humani Corporis Fabrica* not only opened a new chapter in the study of life sciences, but also laid the cornerstone of modern medicine. In 1616, William Harvey described the structure of the human heart and established the theory of blood circulation. In the 1660s, Marcello Malpighi discovered that the respiratory system of small animals has a higher surface-to-volume ratio than that of higher animals. In 1665, Robert Hooke published *Micrographia* and coined the term 'cell' to describe the basic unit of life. In 1683, Antonie van Leeuwenhoek discovered bacteria. In 1688, he gave detailed description of red blood cells. In light of these examples, we can see that discoveries in biology have improved our understanding of the microscopic world.

The 17th century was an age of grand discoveries for the study of biological species. In 335 BC, Aristotle conducted research and provided detailed description for over 500 animal species. He coined the term "animal" and wrote the first book on zoology, which dictated the academic field for over a millennium. In the 1600s, the number of plant species known to science was approximately 6000. Then in the 17th century, scientists discovered 12000 new plant species [1], twice the total number found over the last 2000 years. They also found quite a number of new animal species, so evidently the period was an age of grand discoveries for the study of biological species.

Owing to the large number of new species being found, there was a growing need of a self-consistent and rational classification system. In 1735, Carl Linnaeus published *Systema Naturae* (*Natural System*) and proposed a new system for classifying plants according to their reproductive organs. Although subconsciously Linnaeus appeared to go against evolutionary views, his classification system ended up pushing new ideas forward. From 1749 to 1788, Georges-Louis Leclerc de Buffon published the multi-volume *Histoire Naturelle* (*Natural History*) and proposed the radical view that similar organisms may have a common ancestor. He was the first scholar to treat the origin-of-species problem in a scientific spirit. In 1785, James Hutton published his theory on sedimentary rocks and biological fossils. Subsequently, the study of fossils became a scientific discipline and an important basis for understanding biological systems, especially prehistoric systems. In 1801, Jean-Baptiste Lamarck published *Système des Animaux sans Vertebres* (*System for Invertebrates*) and proposed the first truly cohesive theory of evolution. In 1809, he published *Philosophie zoologique* (*Zoological Philosophy*), which outlined his concept of Lamarckism and coined the term 'biology' for the first time [1]. In 1831, Charles Darwin began his five-year voyage around the world, investigating geology and making natural history collection. After extensive investigations, he established the concept of biological evolution. In 1859, Darwin published *On the Origin of Species* [1], grouping various evolutionary ideas together into one ambitious but convincing theory. It can be said that, without the discovery of biological species and Darwin's expedition around the world, we would not have the theory of evolution as we know it today.

The advent of cell theory in the 19th century triggered a new area in modern medicine, known as experimental medicine. In 1831, the Scottish botanist Robert Brown discovered the nucleus. In 1838, Matthias Jakob Schleiden published a paper titled *Beiträge zur Phytogenesis* (*Contributions to Phytogenesis*), where he stated that "all plants, no matter how complex, are composed of cells" [1]. In 1839, Theodor Schwann published a paper titled *Microscopic Investigations on the Accordance in the Structure and Growth of Plants and Animals*, where he concluded that "all living things are composed of cells". The theory broke the boundary between animals and plants. In 1858, Rudolf Virchow published *Cellular Pathology*, where he pointed out that "all cells come from other cells" and that "all diseases involve changes in normal cells". In 1875, Eduard Adolf Strasburger published *Zellbildung und Zelltheilung* (*Cell Formation and Cell Division*), where he pointed out that "all cells come from pre-existing cells" and that "all nuclei come from other nuclei". From then on, the cell theory was no longer a hypothesis. It was the guiding force of life sciences.

As microscope technology matured in the second half of the 19th century, life science ushered in the era of microorganisms (Table 2). It was a revolutionary period for microbiology research, one that was comparable to the theory of evolution. In 1856, Louis Pasteur discovered that "fermentation is caused by the growth of microorganisms". In 1859, he disproved the theory of spontaneous generation. From 1860 to 1880, Pasteur made huge contributions to wine pasteurization, silkworm diseases and infectious diseases just by one word "microorganism". He proposed the mechanism by which disease was transmitted. From 1870 to 1890, Robert Koch developed new theories and techniques in bacteriology (e.g., preparing pure culture and staining). He proposed and validated the idea that "an organism can be isolated in every case of a disease". He also formulated Koch's postulates, which say that, to establish that an organism is the cause of a disease: it must be found in all cases of the disease examined; it must be prepared and maintained in a pure culture; it must be capable of producing the original infection; and it must be retrievable from an inoculated animal and cultured again. Up to date, Koch's postulates remain the iron laws and gold standards of pathology. In 1890, Ilya Ilyich Mechnikov discovered phagocytes and phagocytosis, as well as proposed the cellular theory of immunity. In 1890, Emil Adolf von Behring discovered a serum therapy against diphtheria and tetanus, as well as proposed the concept of humoral immunity. These discoveries led human beings landing the absolutely new invisible world which species number could be comparable with those of the traditional, visible worlds of animals and plants, defended human rationality against ignorance and Christian supremacy, and triggered a revolution in modern medicine, known as the antibiotic revolution.

The 20th century has been described as the century of the gene [2]. The development of genetics happened so quickly, like a fairy tale in life sciences. In 1900, Hugo de Vries, Karl Correns and Erich von Tschermak independently rediscovered Mendel's genetic laws. From 1910 to 1930, Thomas Hunt Morgan discovered the idea of genetic linkage and produced the first linkage map of the fruit fly *Drosophila*. In his books *The Physical Basis of Heredity* and *the Theory of the Gene*, he provided the first working theory of heredity. In 1928, Frederick Griffith [1] discovered a transforming factor in *Streptococcus pneumoniae*. In 1944, Oswald Avery [1] used biochemical methods to confirm that the transforming factor was, in fact, DNA. In 1937, the European pioneer of structural biology William Astbury proposed a structural model for DNA. He took X-ray diffraction photographs of DNA, showing that its structure is strong, rigid and fibrous. In 1951, Maurice Wilkins and Rosalind Franklin [1] obtained high-quality X-ray diffraction photographs of DNA, indicating that its structure is most likely to be a right-handed double helix. In 1952, Alfred Hershey and Martha Chase used isotope labelling method to prove that the genetic material of bacteriophages is DNA, not protein. In the same year, Erwin Chargaff [1]

**Table 2** Discovery Timeline of pathogenic microorganisms

| Year | Pathogen | Disease caused | Origin | Discovered by |
|---|---|---|---|---|
| 1877 | *Bacillus anthracis* | Anthrax | Germany | Robert Koch |
| 1878 | *Staphylococcus* | Pyogenesis | Germany | Robert Koch |
| 1879 | *Mycobacterium leprae* | Leprosy | Sweden | Hansen Armaner |
| 1879 | *Neisseria gonorrhoeae* | Gonorrhoea | Germany | Neisser A. L. S. |
| 1880 | *Staphylococcus aureus* | Toxic shock syndrome | Scotland | Alexander Ogston |
| 1880 | *Salmonella typhi* | Typhoid | Germany | Eberth C. J. |
| 1881 | *Streptococcus* | Pyogenesis | Scotland | Ogston A. |
| 1882 | *Mycobacterium tuberculosis* | Tuberculosis | Germany | Robert Koch |
| 1884 | *Corynebacterium diphtheriae* | Diphtheria | Germany | Edwin Klebs & Friedrich Loffler |
| 1884 | *Vibrio cholerae* | Cholera | Germany | Robert Koch |
| 1884 | *Clostridium tetani* | Tetanus | Germany | Nicolaier A. |
| 1885 | *Escherichia coli* | Diarrhoea | Germany | Ehrlich Paul |
| 1886 | *Streptococcus pneumoniae* | Pneumonia | Germany | Fraenkel Karl & Louis Pasteur |
| 1887 | *Neisseria meningitides* | Epidemic cerebrospinal meningitis | Germany | Weickselbaum Antou |
| 1888 | *Salmonella enteritidis* | Food poisoning | USA | Gaertner A. A. H. |
| 1892 | *Clostridium perfringens* | Gas gangrene | USA | Welch W. H. |
| 1892 | *Haemophilus influenzae* | Meningitis and pneumonia | Germany | Pfeffer Wilhelm |
| 1894 | *Yersinia pestis* | Plague | Germany | Independently discovered by Kitasato S. & Yersin A. J. E. |
| 1896 | *Clostridium botulinum* | Botulism | Belgium | van Ermengem E. M. P. |
| 1896 | *Salmonella paratyphi* | Paratyphoid | France | Achard Charles |
| 1898 | *Shigella dysenteriae* | Dysentery | Japan | Shiga K. |
| 1911 | *Treponema pallidum* | Syphilis | Japan | Hideyo Noguchi |
| 1906 | *Bordetella pertussis* | Pertussis | France | Bordet J. & Gengou O. |
| 1909 | *Bartonella bacilliformis* | Bartonella disease | South America | Albert Barton |
| 1973 | *Rotavirus* | Infant diarrhoea | Australia | Bishop R. F. |
| 1975 | *Hepatitis A virus* | Hepatitis A | Germany | Stephen M. Feinstone |
| 1976 | *Ebola virus* | Ebola hemorrhagic fever | Africa | Bowone & Pattyn |
| 1977 | *Hantaan virus* | Hemorrhagic fever with renal syndrome | Korea | Lee Ho-wang |
| 1977 | *Legionella* | Legionnaires' disease | USA & Philippines | McDade |
| 1978 | *Hepatitis D virus* (HDV) | Hepatitis D | Italy | Mario Rizzetto |
| 1981 | *Human T-lymphotropic virus 1* (HTLV-1) | T- cell lymphoma, leukaemia | USA | Bernard Poiesz & Francis Ruscetti |
| 1982 | *E. coli. O157*, H7 | Hemorrhagic colitis | USA | Riley |
| 1982 | *Human T-lymphotropic virus 2* (HTLV-2) | Hairy cell leukaemia | Japan | Kalyanaraman V. S. |
| 1982 | *Borrelia burgdorferi* | Lyme disease | USA | Burdorfer |
| 1982 | *Enrterocytozoon bieneusi* | Intractable diarrhoea | USA | Gourley |
| 1983 | *Human immunodificiency virus* (HIV) | AIDS | USA & France | Robert Gallo & Luc Montagnier |
| 1983 | *Helicobacter pylori* | Chronic gastritis, peptic ulcer | Australia | Barry J. Marshall & Warren J. Robin |
| 1986 | *Human herpesvirus 6* (HHV-6) | Early childhood roseola | USA | Robert C. Gallo |
| 1983 | *Hepatitis E virus* (HEV) | Hepatitis E | Russia | Balayan |
| 1989 | *Hepatitis C virus* (HCV) | Hepatitis C | USA | Michael Houghton |
| 1989 | *Ehrlichia chafeensis* | Human ehrlichiosis | USA | Burt E. Anderson |
| 1990 | *Human herpesvirus 7* (HHV-7) | Heat rash, CNS infection | USA | Niza Frekel |
| 1991 | *Gunarito virus* | Venezuelan hemorrhagic fever | Venezuela | Salas R. |
| 1991 | *Encephalitozoon hellem* | Meningitis, diffuse lung diseases | USA | Didier E. S. |
| 1991 | *A new species of Babesia* | Atypical babesiosis | USA | Quick R. E. |
| 1992 | *Vibrio cholerae* | Cholera | Asia | Filippo Pacini |
| 1993 | *Sin Nombre virus* | Adult respiratory distress syndrome | USA | Terry Yates |
| 1994 | *Sibia virus* | Brazilian hemorrhagic fever | Brazil | Barry M. |
| 1995 | *Human herpevirus 8* (HHV-8) | AIDS-related Kaposi's sarcoma | USA | Enrique A. Mesri |
| 1995 | *Hepatitis G virus* (HGV) | Hepatitis D | USA | Simons J. N. |
| 1996 | *Prion* | New Creutzfeldt–Jakob disease | USA | Stanley B. P. Prusiner |
| 1997 | *Transfusion Transmitted virus* (TTV) | Post-transfusion hepatitis | Japan | Nishizawa T. |
| 1997 | *Avian influenza virus* (H5N1) | Influenza | Hong Kong | de Jong |
| 1998 | *West Nile virus* | Nile fever | Uganda | Smithburn K. C. |
| 1999 | *Nipah virus* | Encephalitis | Malaysia | Chua K. B. |
| 2003 | *SARS-CoV* | Sever acute respiratory syndrome | China | |
| 2003 | *H9N2* | Influenza | China | |
| 2005 | *Human bocavirus* | Pneumonia | Sweden | Allander |
| 2010 | *Severe fever with thrombocytopenia syndrome bunyavirus* | Fever with thrombocytopenia syndrome | China | Xuejie Yu |

showed that DNA has percentage base pair equality (%A=%T and %G=%C). Based on the finding, James Watson and Francis Crick [3] discovered the DNA double helix in 1953. They suggested that the specific pairing of bases may be the replicate mechanism for genetic materials, and that the linear sequence of bases may be the code for carrying genetic information. These revolutionary conjectures represent the essence of the DNA double helix. They have made a huge splash in life sciences and took the study of molecular biology to new high. In addition, they have uncovered secrets of the biological world and made genetic engineering one of the most successful scientific disciplines. The 20th century was, without a doubt, the golden era of life sciences, especially the age of grand discoveries.

In 1955, Severo Ochoa demonstrated the first synthesis of RNA. In 1957 Arthur Kornberg isolated the first DNA polymerase and synthesized the first artificial DNA. The finding played a key supportive role in the explanation of the genetic code (Nobelprize.org). In 1955, Frederick Sanger [4] determined the amino acid sequence of bovine insulin. In 1958, John Kendrew and co-workers [5] determined the three-dimensional structure of myoglobin; and Francis Crick [6] published the groundbreaking paper *On Protein Synthesis* where he proposed his 'sequence hypothesis', discussing the relation between the sequence of bases in DNA and the sequence of amino acids in proteins, as well as the 'central dogma' (from DNA to RNA to protein). In 1960, François Jacob and co-workers [7] put forward the concepts of mRNA and 'operon', detailing the regulatory mechanisms that underlie genetic information transfer and protein synthesis. In 1961, Marshall Nirenberg and Heinrich Matthaei [8] cracked the first codon of the genetic code. In 1970, David Baltimore [9], Howard Temin and Satoshi Mizutani [10] discovered the reverse transcriptase and expanded the 'central dogma', with molecular biology being well-established.

Molecular biology was largely inspired by the reductionist approach of the 1970s, which proved to be extremely successful and helped to unravel many of the basic molecular and cellular processes. Two groups, one led by Stuart Linn and Werner Arber [11] and the other led by Hamilton Smith and Kent Wilcox [12], independently discovered two useful 'tools' for cutting and ligating DNA: the restriction enzyme and the modification enzyme. In 1970, Daniel Nathans used these enzymes to demonstrate the cleavage of DNA *in vitro*. In 1971, Paul Berg used these enzymes to create the first recombinant DNA. In 1976, Yuet Wai Kan reported the first case of DNA diagnosis in monogenic diseases. In 1977, John Michael Bishop [13] discovered the oncogene *src*. In 1975, Frederick Sanger and co-workers [14] discovered a DNA sequencing method and used it to decode the whole genome of bacteriophage φX174 two years later. In 1978 and 1981, Sidney Altman and Thomas Cech independently discovered the catalytic abilities of RNA. In 1979, David Goeddel successfully used genetic engineering to coax bacteria into mass-producing insulin. In 1982, Richard Palmiter and Ralph Brinster produced transgenic mice, supermice (that express a growth hormone). In 1985, Kary Mullis and her team [15] developed PCR technology for amplifying DNA *in vitro*. The genetic techniques have now become a series of indispensable magic tools in gene sequencing, genetic recombination, DNA diagnosis, genetic engineering, gene transfer and gene amplification.

Evidently, there had been many continuous and overlapping periods of profound discoveries in the history of contemporary life sciences. At every stage of the game, there would be one or more leading scientists showing great foresights, turning things around and opening up new avenues of research. These great scientists could pierce through the dark sky and guide the discipline forward into a new age of grand discoveries.

## 3 The age of 'omics'

Life forms are the most complex physical systems known thus far. Take the human body as an example. From a reductionist point of view, as we move from organs to tissues and from cells to molecules, our research subject gets divided into tens or millions individual pieces. Moreover, the number of interactions among these individual pieces grows exponentially. From a systems theory point of view, we need to study not only the multi-level dynamical changes of the human biology, but also the complex physical and chemical factors of the human environment, the numerous symbiotic microbes of the human ecology, and the various psychosocial factors of the human society. Our awareness of life is like Zeno's circle: when our circle of knowledge becomes greater, our contact with the unknown world also becomes greater. Life may look like a magnificent and poetic painting in the eyes of ordinary people. Under the microscope, however, this painting is nothing but pixels, lines and blocks of various shades and colours. In this new age of life sciences, scientists are puzzling over how to connect dots, lines, planes and solids to make something artistic, something that is neither *The Blind Men and the Elephant* nor Giuseppe Arcimboldo's *Vertumnus* (Figure 1). Lifeomics is like a Russian doll. Just when you think you understand the situation, another mystery of life emerges. The reductionist approach seeks to understand living systems through the study of their constituent parts—from total syntheses to regulatory mechanisms of various biomacromolecules. By taking the reductionist approach to the extreme, we can get to the bottom of the problem, turn it into a theory and make the most of our time. The holistic approach seeks to integrate all constituents into one single system, which could turn something simple into something extraordinary. Only a 'grand master' who understands the magic of this approach can push the discipline to new high. The reductionist approach begins with 'ome' and ends with

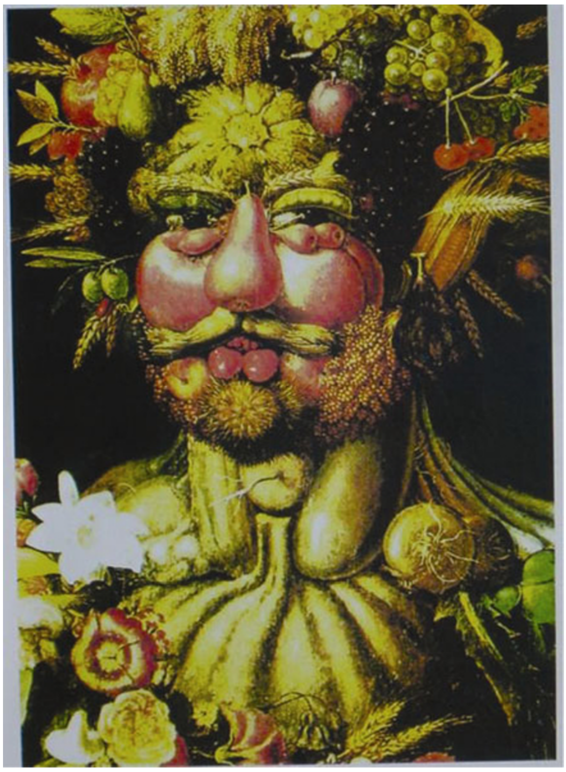

**Figure 1** Giuseppe Arcimboldo's *Vertumnus*.

'o,m,e' ('O', 'm' and 'e' are respectively to 'ome' what 'subset' is to 'set' in mathematics.), whereas the holistic approach begins with 'o,m,e' and ends with 'ome'. Genomics, RNAomics, proteomics and metabolomics are examples of the latter. This is why lifeomics, which uses unique epistemology and methodology, quickly became the thrust of life sciences.

The development of omics has led to many great discoveries in life sciences since the late 20th century. In 1953, Watson and Crick proposed the double helix structure of DNA and said, "the precise sequence of the bases is the code which carries genetic information". In 1958, Sanger from the same laboratory established the method for determining amino acid sequences of protein (http://www.nobelprize.org/nobel_prizes/chemistry/laureates/1958/sanger.html). In the 1970's, he established the method for analysing DNA sequences [16]. Because of these two achievements, Sanger won the Nobel Prize in Chemistry twice. As the king of DNA sequencing, Sanger turned our dreams of reading and writing genetic information into a reality. He made DNA sequencing a part of life. Through DNA sequencing, scientists could integrate biotechnology with information technology. In 1986, the first generation of fluorescence-based DNA sequencers with automated analysis was born. Since then, our DNA sequencing capacity has been growing exponentially. Our current daily data output is in the Gb range [17], a rate that could rival Moore's Law. Protein sequencing technologies based on mass spectrometry represent another force to be reckoned with. Biological mass spectrometry, for example, was awarded the Nobel Prize in Chemistry in 2002. At that time, scientists could only identify several hundreds of proteins in a given sample. Today, biological mass spectrometry could identify several thousands of proteins in a single sample, which is about the size of a transcriptome. These high-throughput sequencing technologies are set to open up new avenues of research and trigger an age of grand discoveries in lifeomics.

The US government launched an international scientific research project, dubbed the Human Genome Project (HGP), four years after the birth of the first DNA sequencer [18]. Since then, countries like Germany, Japan, UK, France and China have joined the project. When the project first began, there were only 100000 bases pairs in all of the human genes known. Ten years later, scientists have not only sequenced the 3 billion base pairs that make up the human genome, but also the genomes of *Saccharomyces* (budding yeast), *Escherichia coli*, *Mycobacterium tuberculosis*, *Treponema pallidum*, *Nematoda* (nematode), *Drosophila* (fruit fly), *Arabidopsis thaliana* (mustard plant), *Oryza sativa* (rice), *Mus musculus* (mouse), *Plasmodium*, *Anopheles* (mosquito), as well as several other important pathogens and model organisms. The project has triggered a great number of follow-up projects, for example, the International HapMap Project in 2002, the 1000 Genome Project in 2008, the 10000 Microbial Genomes Project in 2009, as well as the 1000 Plants and Animals project and the Genome 10K project in 2010. A decade has passed since the publication of the first human genome (October 2011), but we have already resolved the genomes of over 1200 biological species. The National Center for Biotechnology Information (NCBI) holds 2.08 Pb (1 Pb=$10^{15}$ b) of genomic data as of 14 June 2012, 10 billion times the amount when the project first started. Chinese scientists, in particular, have contributed more than 0.64 Pb of data, which accounts for 30% of the total. They made outstanding contributions in three particular areas: genetic information, gene function and medical genetics. In terms of genetic information, although we can only interpret some and not all of the information, the genome sequencing has enabled us to read into the evolutionary process that lasted for hundreds of millions of years. Having the genetic information (the blueprint of life) of thousands of species represents a huge advantage, but this is only the tip of the iceberg. We expect to see more discoveries not only in the number of species, but also in the realisation of richer genomes through whole or single-cell genome and transcriptome sequencing. These breakthroughs will give us the ability to synthesize and create life. In terms of gene function, HGP has already identified more than 20000 protein-coding genes, when scientists only knew a fraction of these genes just a century ago. The findings will help unveil the mysteries behind the human genome, transcriptome and proteome, as well as make a splash in comparative genomics and animal genomics. Studies have shown that less than 3% of the human genome is protein-coding genes. Therefore, following the surprising discovery of dark matter and dark energy in physics, we might find astonishing 'dark information' in life sciences. DNA sequencing technology is now within reach of ordinary people thanks the rapid rise in capacity and the sharp decline in price over the past 20 year. It has become a common tool in biological research and clinical diagnosis. In the area of medical genetics, for an example, we could take genome-wide association study (GWAS). GWAS has existed only for less than ten years, and during this period of time we have studied more than 700 diseases and discovered more than 5000 disease-causing genes and susceptibility loci, ten times the number found 100 years ago. Although China was a late starter in genomics research, the country's achievements in the field are still remarkable (Table 3). This is in line with what Watson said ten years ago, "all biology in the future will start with the knowledge of genomes and proceed hopefully".

Genome sequencing is like the winds of a rising storm. To better understand the human genome, we must part the clouds and swirl things around. In the month that the draft of the human genome was published, the launch of the Human Proteome Organization (HUPO) was announced. In the following year, the Human Proteome Project (HPP) was initiated. Given the variety of proteins (at least 100 times the number of genes), the huge dynamic range of protein abundance, the diversity of post-translational modifications, the complex issue of space-time specificity and the great number of interactions between the proteins, HPP launched a series of pilot projects during its initial phase. In 2002, HPP launched the Human Liver Proteome Project (HLPP) and the Human Plasma Proteome Project (HPPP). After this, HPP launched the Human Brain Proteome Project (HBPP), Human Kidney and Urine Proteome Project (HKUPP) and Human Cardiovascular Initiate (HCVI). It has also launched Proteomics Standards Initiative (PSI), Human Antibody Initiative (HAI) and Disease Biomarkers Initiative (Table 4). In 2005, HPPP generated a core dataset of 3020 proteins, the first human body fluid proteome of its kind. In 2010, HLPP released a core dataset of 6788 proteins, the first human organ proteome of its kind [51]. Over half of these proteins were identified in the human liver for the first time. Moreover, about a quarter of them were newly discovered at the protein level. In 2009, Chinese scientists discovered over 1000 acetylated proteins by using high-throughput protein analysis and studying metabolic enzymes across different species [52]. In addition, they identified the role of acetylation in the regulation of metabolic enzymes. In 2012, Chinese scientists used bacteria, yeast, nematode, fruit fly, mouse and human as research subjects and, through large-scale quantitative proteomic data analyses, found three universal patterns in the overall distribution of protein abundance [53]: protein abundance is positively correlated with the protein's origination time or sequence conservation during evolution; protein abundance is negatively correlated with the protein's domain number and positively correlated with domain coverage in protein structure; and proteins that act on material conversion and transportation are more abundant than those that act on information modulation. The findings demonstrated that the large-scale dataset of omics could be the resource for the discovery of the laws of life. Other organ/tissue proteome projects have also generated 1000–3000 proteins. With increasing technological capabilities in recent years, biological mass spectrometry can now identify 7000–9000 proteins in a cell line sample, with gene expression coverage reaching 60%–90%. Research on protein interaction networks has also made amazing progress. In 2005, two research groups in Germany and USA independently established human protein interaction networks featuring 3169 pairs and 2,754 pairs of proteins [54]; in 2011, China scientists established a human liver protein interaction network that encompasses 3484 interactions among 2582 proteins [55]. In the three studies we just mentioned, there had only been 54 cases of overlapping protein interactions. The number suggests that more than 90% of protein interaction network is still unknown and waiting to be discovered. In 2011, research on human endogenous protein complexes has identified more than 11000 products of gene expression, which accounts for about 50% of the total. In addition, HAI released the 7th version of its database, which covers 10118 products of coding genes. In 2009, OVA1, the first proteomics-discovered cancer biomarker discovered by Daniel Chan and his team received approval from the USA Food and Drug Administration (FDA) for detecting ovarian cancer (http://www.fda.gov/NewsEvents/Newsroom/Press-An-nouncements/ucm182057.htm). The event signals the advent of more great discoveries and clinical applications in proteomics.

Genomics and proteomics are the two most active areas within the field of lifeomics. After the completion of HGP, it was found that protein-coding sequences make up only about 2% of the human genome [56]; among the non-coding DNA and RNA sequences, about 1/4 are tRNA and rRNA, and 3/4 are snRNA, snoRNA, shRNA, microRNA and lncRNAs (long non-coding RNAs), which have important roles in chromatin remodelling, transcriptional regulation and translational regulation [57], affecting biological development, cell proliferation, cell differentiation, as well as the development of cancer, metabolic diseases and viral diseases. Since the advent of 'RNAomics' in 2000, more than 35000 non-coding RNA have already been discovered in mammals. The mutual regulation between DNA, mRNA, protein and non-coding RNA has attracted wide attention from the community. The technology RNAi won the 2006 Nobel Prize in Physiology and Medicine only eight years after it was discovered. Such case is rare in the Nobel Prize history. Coincidentally, systems biologists have taken an interest in 'glycomics', which studies the structure and recognition molecules of oligosaccharides, polysaccharides

**Table 3** GWAS conducted by Chinese scientists

| Year of publication | Disease studied | Research group | Reference |
|---|---|---|---|
| 2009 | Psoriasis | Xuejun Zhang et al. | [19] |
| 2009 | Systemic lupus erythematosus | Xuejun Zhang et al. | [20] |
| 2009 | Leprosy | Furen Zhang et al. | [21] |
| 2010 | Nasopharyngeal cancer | Yixin Zeng et al. | [22] |
| 2010 | Vitiligo | Xuejun Zhang et al. | [23] |
| 2010 | Hepatocellular carcinoma | Fuchu He & Gangqiao Zhou et al. | [24] |
| 2010 | Oesophageal cancer | Lidong Wang et al. | [25] |
| 2010 | Agronomic traits of *indica* rice | Bin Han et al. | [26] |
| 2010 | Polycystic ovary syndrome | Zijiang Chen et al. | [27] |
| 2011 | Coronary artery disease | Qing Wang et al. | [28] |
| 2011 | Oesophageal cancer | Dongxin Lin et al. | [29] |
| 2011 | Atopic dermatitis | Xuejun Zhang et al. | [30] |
| 2011 | Lung cancer | Hongbing Shen et al. | [31] |
| 2011 | Graves' disease | Huaidong Song et al. | [32] |
| 2011 | Gastric cancer | Hongbing Shen et al. | [33] |
| 2011 | Schizophrenia | Lin He et al. | [34] |
| 2011 | Schizophrenia | Dai Zhang et al. | [35] |
| 2011 | Leprosy | Furen Zhang et al. | [36] |
| 2011 | Flowering time and grain yield of rice | Bing Han et al. | [37] |
| 2011 | Pancreatic cancer | Dongxin Lin et al. | [38] |
| 2011 | Ankylosing spondylitis | Jieruo Gu et al. | [39] |
| 2011 | IgA nephropathy | Xueqing Yu et al. | [40] |
| 2011 | Non-obstructive azoospermia | Jiahao Sha et al. | [41] |
| 2012 | Kawasaki disease | Jer Yuarn Wu et al. | [42] |
| 2012 | Coronary artery disease | Dongfong Gu et al. | [43] |
| 2012 | Polycystic ovary syndrome | Zijiang Chenet al. | [44] |
| 2012 | Thyrotoxic periodic paralysis | Annie Wai Chee Kung et al. | [45] |
| 2012 | Oesophageal cancer | Dongxin Lin et al. | [46] |
| 2012 | Prostate cancer | Yinghao Sun et al. | [47] |
| 2012 | Hepatocellular carcinoma | Long Yu et al. | [48] |

**Table 4** Progress of the Human Proteome Project

| Year of initiation | Sub-project | Dataset | Release year |
|---|---|---|---|
| 2002 | Human Liver Proteome Project | ProteomeView, 6847 proteins (6788 [51]) | 2010 |
| 2002 | Human Plasma Proteome Project | Human Plasma PeptideAtlas, 1929 proteins (3020 [49]) | 2005 |
| 2003 | Human Brain Proteome Project | 1832 (human)/792 (mouse) proteins | 2010 |
| 2003 | Proteomics Standardization Initiative | Protein information, mzML<br>Molecular interaction, PAR/MIAPAR/PSICQUIC<br>Protein separation, MIAPE-GEL/MIAPE-CC/MIAPE-CE | 2010 |
| 2005 | Human Kidney and Urine Proteome Project | Human Kidney and Urine Proteome Project, 3679 proteins; the final standards for non-protein urine and urine proteome | 2009 |
| 2005 | Human Antibody Initiative | The 10th edition of database encompasses 14079 coding genes | 2012 |
| 2005 | Human Disease Glycomics/Proteome Initiative | Comparison of full-spectrum analysis methods for O-glycosylation; full-spectrum analysis of glycoproteins in cancer cells | 2010 |
| 2005 | Mouse Model of Human Disease | More than 1400 mouse secreted proteins have been identified | 2006 |
| 2006 | Human Cardiovascular Initiative | 1333 proteins with more than 10000 GO annotations; more than half of which are from human data | 2009 |
| 2007 | Proteome Biology of Stem Cell Initiative | Stem cell markers, stem cell signalling pathways and stem cells & diseases | 2009 |
| 2009 | Disease Biomarkers Initiative | Markers for cancer, cardiovascular disease and pulmonary disease | 2010 |
| 2010 | Model Organism Proteomes | Advances in model organisms | 2010 |
| 2010 | Chromosome-based Human Proteome Project | J Proteome Res, Publication Date (Web): January 11, 2013 | 2013 |

and various sugar chains, as well as 'metabolomics', which studies the endogenous metabolites and their responses to external stimuli. Much progress has been made over the past ten years, and these omics are becoming a major force in life sciences.

Like the Chinese saying goes, "when spring comes, all flower blossoms". Different omics have made varying levels of contributions to life sciences. In 2005, HPP initiated the Human Disease Glycomics/Proteome Initiative (HGPI), a project that aims to identify useful glycobiomarkers for detecting life-style diseases by integrating proteomics with glycomics. In 2011, HPP formed Chromosome-based Human Proteome Project (Ch-HPP), a joint project that uses proteomics technology to determine the gene product of each chromosome. Ch-HPP brings together the best talents from HPP and HGP. Like the Chinese saying goes, "life does not discriminate for benevolence". We should treat all 'omics' as equally important. And lifeomics, which integrates genomics and RNAomics with proteomics, glycomics and metabolomics, has taken shape and is now ready to take on any challenges.

Looking back at the history of natural sciences, especially in life sciences, we can draw an analogy between the age of grand discoveries and nuclear fusion. We understand that only through fusion can we release large amounts of energy. However, to obtain enough energy to trigger the fusion process, we need nuclear fission.

Therefore, HGP was not just a beginning. It was the fission point that triggered the development of various omics. Lifeomics, which encompasses all these omics, is set to achieve something big. To our knowledge, projects utilizing a similar research approach have only appeared twice in human history: once in the Manhattan project and once in the Apollo program. In both cases, the scientific discoveries made have had far-reaching impact on our society.

With life come organic, as the biological world is the culmination (organization) of, for and over the physical world (from inorganic to organic to biological). With life also come human cognition, and this is the magic that connects the biological and the physical world. Because of this, we trust that life science is going to be the epitome and assemble of all disciplines of natural sciences. Furthermore, lifeomics is the backbone for life science. Thus, it is going to be the rising star of natural sciences in the coming future.

*I thank the Academic Divisions of the Chinese Academy of Sciences, Science China Press, Forum on Frontiers of Science and Technology and the standing committee of the Academic Division of Life Sciences and Medicine for their support on the 7th forum on frontiers of science and technology:Lifeomics and Translational Medicine Symposium. I also thank Qiang BoQin and Zeng YiXin for co-organizing this forum; Shi YunYu, Chen KaiXian, Zhao GuoPing, He Lin, Zeng YiXin, Yang HuanMing, Chen RunSheng, Cheng Jing, Zhang XueMin, Wu JiaRui, Wang Jun, Han JingDong, Zhu WeiMin and Li Ling for giving their excellent reports; Wang DongGen, Zhen Bei, Fu Min, Shi YuKun and Zhao Liang for ensuring the smooth-running of the forum; Wu ZhenYu, Zhang XueLi, Wang Yan, Cao WuChun, Zhang HongXing, Sun Wei and Yang Dong for drafting the detailed records in the forum. This work is funded by China-Australia Joint Science and Technology Commission (2010DFA31260) and China-Canada Joint Health Research Initiative (81010064).*

1 Wu G S. The evolution of science (in Chinese). Changsha: Hunan Science and Technology Press, 1995
2 Gelbart W M. Databases in genomic research. Science, 1998, 282: 659–661
3 Watson J D, Crick F H C. Genetical implications of the structure of deoxyribonucleic acid. Nature, 1953, 171: 964–967
4 Ryle A P, Sanger F, Smith L F, et al. The disulphide bonds of insulin. Biochem J, 1955, 60: 541–556
5 Kendrew J C, Bodo G, Dintzis H M, et al. A three-dimensional model of the myoglobin molecule obtained by X-ray analysis. Nature, 1958, 181: 662–666
6 Crick F H C. On protein synthesis. Symp Soc Exp Biol XII, 1958, 12: 138–163
7 Jacob F, Perrin D, Sanchez C, et al. The operon, a group of genes with expression coordinated by an operator. C R Acad Sci Paris, 1960, 250: 1727–1729
8 Nirenberg M W, Matthaei J H. The dependence of cell-free protein synthesis in *E. coli* upon naturally occurring or synthetic polyribonucleotides. Proc Natl Acad Sci USA, 1961, 47: 1588–1602
9 Baltimore D. RNA-dependent DNA polymerase in virions of RNA tumour viruses. Nature, 1970, 226: 1209–1211
10 Temin H M, Mizutani S. RNA-dependent DNA polymerase in virions of Rous sarcoma virus. Nature, 1970, 226: 1211–1213
11 Linn S, Arber W. Host specificity of DNA produced by *Escherichia coli*, X. *In vitro* restriction of phage fd replicative form. Proc Natl Acad Sci USA, 1968, 59: 1300–1306
12 Smith H, Wilcox K W. A restriction enzyme from *Hemophilus influenzae*. I. Purification and general properties. J Mol Biol, 1970, 51: 379–391
13 Stehelin D, Fujita D J, Padgett T, et al. Detection and enumeration of transformation-defective strains of avian sarcoma virus with molecular hybridization. Virology, 1977, 76: 675–684
14 Sanger F, Air G M, Barrell B G, et al. Nucleotide sequence of bacteriophage phi X174 DNA. Nature, 1977, 265: 687–695
15 Saiki R, Scharf S, Mullis K B, et al. Enzymatic amplification of beta-globin genomic sequences and restriction site analysis for diagnosis of sickle cell anemia. Science, 1985, 230: 1350–1354
16 Sanger F, Coulson A R. A rapid method for determining sequences in DNA by primed synthesis with DNA polymerase. J Mol Biol, 1975, 94: 441–448
17 Gonzaga-Jauregui C, Lupski J R, Gibbs R A, et al. Human genome sequencing in health and disease. Annu Rev Med, 2012, 63: 35–61
18 Lander E S, Liton L M, Birren B, et al. Initial sequencing and analysis of the human genome. Nature, 2001, 409: 860–921
19 Zhang X J, Huang W, Yang S, et al. Psoriasis genome-wide association study identifies susceptibility variants within LCE gene c luster at 1q21. Nat Genet, 2009, 41: 205–210
20 Han J W, Zheng H F, Cui Y, et al. Genome-wide association study in a Chinese Han population identifies nine new susceptibility loci for systemic lupus erythematosus. Nat Genet, 2009, 41: 1234–1237
21 Zhang F R, Huang W, Chen S M, et al. Genomewide association study of leprosy. N Engl J Med, 2009, 361: 2609–2618
22 Bei J X, Li Y, Jia W H, et al. A genome-wide association study of nasopharyngeal carcinoma identifies three new susceptibility loci. Nat Genet, 2010, 42: 599–603
23 Quan C, Ren Y Q, Xiang L H, et al. Genome-wide association study for vitiligo identifies susceptibility loci at 6q27 and the MHC. Nat Genet, 2010, 42: 614–618
24 Zhang H, Zhai Y, Hu Z, et al. Genome-wide association study identifies 1p36.22 as a new susceptibility locus for hepatocellular carcinoma in chronic hepatitis B virus carriers. Nat Genet, 2010, 42: 755–758
25 Wang L D, Zhou F Y, Li X M, et al. Genome-wide association study of esophageal squamous cell carcinoma in Chinese subjects identifies susceptibility loci at PLCE1 and C20orf54. Nat Genet, 2010, 42: 759–763
26 Huang X, Wei X, Sang T, et al. Genome-wide association studies of 14 agronomic traits in rice landraces. Nat Genet, 2010, 42: 961–967
27 Chen Z J, Zhao H, He L, et al. Genome-wide association study identifies susceptibility loci for polycystic ovary syndrome on chromosome 2p16.3, 2p21 and 9q33.3. Nat Genet, 2011, 43: 55–59
28 Wu C, Hu Z, He Z, et al. Genome-wide association identifies a susceptibility locus for coronary artery disease in the Chinese Han population. Nat Genet, 2011, 43: 345–349
29 Wu C, Hu Z, He Z, et al. Genome-wide association study identifies three new susceptibility loci for esophageal squamous-cell carcinoma in Chinese populations. Nat Genet, 2011, 43: 679–684
30 Sun L D, Xiao F L, Li Y, et al. Genome-wide association study identifies two new susceptibility loci for atopic dermatitis in the Chinese Han population. Nat Genet, 2011, 43: 690–694
31 Hu Z, Wu C, Shi Y, et al. A genome-wide association stud y identifies two new lung cancer susceptibility loci at 13q12.12 and 22q12.2 in Han Chinese. Nat Genet, 2011, 43: 792–796
32 Chu X, Pan C M, Zhao S X, et al. A genome-wide association study identifies two new risk loci for Graves' disease. Nat Genet, 2011, 43: 897–901
33 Shi Y, Hu Z, Wu C, et al. A genome-wide association study identifies new susceptibility loci for non-cardia gastric cancer at 3q13.31 and 5p13.1. Nat Genet, 2011, 43: 1215–1218
34 Shi Y, Li Z, Xu Q, et al. Common variants on 8p12 and 1q24.2 confer risk of schizophrenia. Nat Genet, 2011, 43: 1224–1227
35 Yue W H, Wang H F, Sun L D, et al. Genome-wide association study identifies a susceptibility locus for schizophrenia in Han Chinese at 11p11.2. Nat Genet, 2011, 43: 1228–1231
36 Zhang F, Liu H, Chen S, et al. Identification of two new loci at IL23R and RAB32 that influence susceptibility to leprosy. Nat Genet, 2011, 43: 1247–1251
37 Huang X, Zhao Y, Wei X, et al. Genome-wide association study of flowering time and grain yield traits in a worldwide collection of rice germplasm. Nat Genet, 2011, 44: 32–39
38 Wu C, Miao X, Huang L, et al. Genome-wide association study identifies five loci associated with susceptibility to pancreatic cancer in Chinese populations. Nat Genet, 2011, 44: 62–66
39 Lin Z, Bei J X, Shen M, et al. A genome-wide association study in Han Chinese identifies new susceptibility loci for ankylosing spondylitis. Nat Genet, 2011, 44: 73–77
40 Yu X Q, Li M, Zhang H, et al. A genome-wide association study in Han Chinese identifies multiple susceptibility loci for IgA nephropathy. Nat Genet, 2011, 44: 178–182
41 Hu Z, Xia Y, Guo X, et al. A genome-wide association study in Chinese men identifies three risk loci for non-obstructive azoospermia. Nat Genet, 2011, 44: 183–186
42 Lee Y C, Kuo H C, Chang J S, et al. Two new susceptibility loci for Kawasaki disease identified through genome-wide association analysis. Nat Genet, 2012, 44: 522–525
43 Lu X, Wang L, Chen S, et al. Genome-wide association study in Han Chinese identifies four new susceptibility loci for coronary artery disease. Nat Genet, 2012, 44: 890–894
44 Shi Y, Zhao H, Shi Y, et al. Genome-wide association study identifies eight new risk loci for polycystic ovary syndrome. Nat Genet, 2012, 44: 1020–1025
45 Cheung C L, Lau K S, Ho A Y, et al. Genome-wide association study identifies a susceptibility locus for thyrotoxic periodic paralysis at 17q24.3. Nat Genet, 2012, 44: 1026–1029
46 Wu C, Kraft P, Zhai K, et al. Genome-wide association analyses of esophageal squamous cell carcinoma in Chinese identify multiple susceptibility loci and gene-environment interactions. Nat Genet, 2012, 44: 1090–1097
47 Xu J, Mo Z, Ye D, et al. Genome-wide association study in Chinese men identifies two new prostate cancer risk loci at 9q31.2 and 19q13.4. Nat Genet, 2012, 44: 1231–1235
48 Jiang D K, Sun J, Cao G, et al. Genetic variants in STAT4 and HLA-DQ genes confer risk of hepatitis B virus-related hepatocellular carcinoma. Nat Genet, 2012, 45: 72–75
49 Omenn G S, States D J, Adamski M, et al. Overview of the HUPO Plasma Proteome Project, results from the pilot phase with 35 collaborating laboratories and multiple analytical groups, generating a core dataset of 3020 proteins and a publicly-available database. Proteomics, 2005, 5: 3226–3245
50 Deutsch E W, Eng J K, Zhang H, et al. Human plasma peptide atlas. Proteomics, 2005, 5: 3497–3500
51 Sun A, Jiang Y, Wang X, et al. Liverbase, a comprehensive view of human liver biology. J Proteome Res, 2010, 9: 50–58
52 Wang Q, Zhang Y, Yang C, et al. Acetylation of metabolic enzymes coordinates carbon source utilization and metabolic flux. Science, 2010, 328: 974
53 Zhong F, Yang D, Hao Y, et al. Regular patterns for proteome-wide distribution of protein abundance across species. PLoS ONE, 2012, 7: e32423
54 Stelzl U, Worm U, Lalowski M, et al. A human protein-protein interaction network, a resource for annotating the proteome. Cell, 2005, 122: 957–968
55 Wang J, Huo K, Ma L, et al. Toward an understanding of the protein interaction network of the human liver. Mol Syst Biol, 2011, 7: 536
56 Pennisi E. Shining a light on the genome's 'dark matter'. Science, 2010, 330: 1614
57 Wapinski O, Chang H Y. Long noncoding RNAs and human disease. Trends Cell Biol, 2011, 21: 354–361

**Open Access** This article is distributed under the terms of the Creative Commons Attribution License which permits any use, distribution, and reproduction in any medium, provided the original author(s) and source are credited.
